# The efficacy of motivational counseling and SMS-reminders on daily sitting time in patients with rheumatoid arthritis: protocol for a randomized controlled trial

**DOI:** 10.1186/s13063-014-0540-x

**Published:** 2015-01-27

**Authors:** Bente Appel Esbensen, Tanja Thomsen, Merete L Hetland, Nina Beyer, Julie Midtgaard, Katrine Løppenthin, Poul Jennum, Mikkel Østergaard, Jan Sørensen, Robin Christensen, Mette Aadahl

**Affiliations:** Nursing and Health Science Research Unit, Glostrup Hospital, University of Copenhagen, Glostrup, Denmark; Copenhagen Center for Arthritis Research (COPECARE), Center for Rheumatology and Spine Diseases, Glostrup Hospital University of Copenhagen, Glostrup, Denmark; Department of Clinical Medicine, Faculty of Health and Medical Sciences, University of Copenhagen, Copenhagen, Denmark; Department of Physio- and Occupational Therapy, Glostrup Hospital, University of Copenhagen, Glostrup, Denmark; Department of Public Health, Faculty of Health and Medical Sciences, University of Copenhagen, Copenhagen, Denmark; DANBIO registry, Center for Rheumatology and Spine Diseases, Glostrup Hospital, University of Copenhagen, Copenhagen, Denmark; Department of Physical Therapy, Musculoskeletal Rehabilitation Research Unit, Bispebjerg and Frederiksberg Hospital, University of Copenhagen, Copenhagen, Denmark; Center for Health Research, Copenhagen University Hospital, Rigshospitalet, Copenhagen, Denmark; Department of Clinical Neurophysiology, Danish Center for Sleep Medicine, Glostrup Hospital, University of Copenhagen, Copenhagen, Denmark; Center for Applied Health Services Research, University of Southern Denmark, Odense, Denmark; Department of Rheumatology, Bispebjerg and Frederiksberg Hospital, Musculoskeletal Statistics Unit, the Parker Institute, University of Copenhagen, Copenhagen, Denmark; Research Center for Prevention and Health, The Capital Region of Denmark, Glostrup Hospital, University of Copenhagen, Copenhagen, Denmark

**Keywords:** Sedentary behavior, Short text message service, Fatigue, Pain, Self-efficacy, ActivPAL, HR-QoL

## Abstract

**Background:**

Patients with RA (Rheumatoid Arthritis) are more sedentary than the general population. Reduction of Sedentary Behaviour (SB) has been suggested as a mean for improvement of health in patients with chronic diseases and mobility problems. Short-term intervention studies have demonstrated that SB can be reduced by behavioural interventions in healthy populations. However, it remains unexplored whether it is valid for patients with RA also.

Therefore, the aim of this trial is to investigate the efficacy of an individually tailored, theory-based motivational counseling intervention on reducing daily sitting time in sedentary patients with RA. Additionally, to explore whether a reduction in daily sitting time is associated with reduced pain and fatigue, self-reported physical function, self-efficacy, improved health-related quality of life (HR-QoL) and cardiovascular biomarker levels, and finally to assess the cost-effectiveness of the intervention.

**Methods/Design:**

For this parallel group randomized trial, 150 patients with RA and at least 5 hours of sitting time per day, will be recruited from a rheumatology outpatient clinic, and block-randomized to the intervention group or the control group receiving usual care. The intervention includes: 1) individual motivational counseling (in total 3 sessions) on reduction of daily sitting time in combination with 2) individual Short Text Message Service (SMS) reminders over a 16-week intervention period. Primary outcome is change in daily sitting time (minutes) from baseline to 16 weeks measured objectively using an ActivPAL® Activity Monitor. Secondary outcomes include fatigue, pain, physical function, HR-QoL, self-efficacy, costs and cost-effectiveness. Furthermore, anthropometric measures will be included as well as measurement of blood pressure and serum lipids. All outcomes are assessed at baseline and repeated after 16 weeks. Follow-up assessments are made at 6 and 18 months post-intervention.

**Discussion:**

The intervention is simple, non-invasive and may be implemented at low costs. If the study confirms the positive results expected, the intervention might be implemented in clinical practice and potentially transferred to other clinical populations.

**Trial registration:**

ClinicalTrial.gov registration number: NCT01969604. Date of registration: 17 October 2013.

## Background

### Background and rationale

Physical inactivity is an established risk factor for chronic disease and premature death [1,2]. In addition to physical inactivity, an increasing number of population-based observational studies [3-5] have shown that sedentary behavior is a distinct and independent risk factor for cardiovascular morbidity and mortality. Patients with rheumatoid arthritis (RA) have an increased risk of cardiovascular disease and premature death [6,7]. The increased risk is partly caused by the chronic inflammatory rheumatic disease itself and partly by traditional risk factors; for example, hyperlipidemia and hypertension, but may also be attributed to physical inactivity [8,9]. In Denmark, 67% of patients with RA do not meet the public health recommendations for daily moderate and vigorous physical activity (MVPA), and similar proportions of physically inactive RA patients are found in Germany (68%) and the United Kingdom (67%) [9].

The everyday life of patients with RA is periodically influenced by increased disease activity (flares) with symptoms such as swelling and stiffness of the joints accompanied by intense pain, which can lead to severe limitations in physical functioning and potential progressive joint destruction [10,11]. Intervention studies in patients with RA have documented a positive effect of exercise on pain and physical functioning [12,13]. However, studies have also demonstrated that exercise and increased activity levels are difficult to maintain over time [12,13] and have identified pain as a main barrier against adaptation and maintenance of a physically active lifestyle in patients with RA [14]. Sedentary behavior has been defined as any waking behavior characterized by an energy expenditure ≤ 1.5 METs (Metabolic Equivalent of Task) while in a sitting or reclining position [15]. Such behavior has become increasingly prevalent in modern society, and recent population studies [16,17] using self-reported questionnaires estimate a mean daily sitting time of 8 to 9 hours, corresponding to 50 to 60% of waking hours. Likewise, a US population-based study among 4,757 adults measured objectively daily sitting time to be on mean 8.44 hours [18]. A few studies have measured sitting time objectively in patients with chronic disease, and these studies generally find that patients with various types of physical disability have higher proportions of sitting time during waking hours; for example, stroke (86 to 88% of waking hours) [19], multiple sclerosis (75 to 85% of waking hours) [20] and Parkinson’s disease (76% of waking hours) [21]. In RA patients, Pioreschi *et al*. found that 71% of waking hours were spent sedentary compared to 62% of waking hours in healthy controls [22]. Objective measures of physical activity and sedentary behavior have been applied in a few other studies in RA patients [23-25]. A review from 2011 suggests that aiming to increase physical activity levels among patients with physical disability should not solely focus on increasing MVPA (moderate and vigorous physical activity) but should also target reduction of sedentary behavior and increase in light intensity activity as this approach may prove feasible for improving health and well-being [26].

A few short-term intervention studies applying objective measures of sitting time in older people [27], and in overweight or obese adults [28,29] have demonstrated that sedentary behavior can be reduced through behavioral intervention [27,28] and that physical activity energy expenditure may be increased by reduction of TV-viewing time [29]. Accordingly, we hypothesize that sedentary behavior can be reduced through lifestyle change in patients with RA.

### Objectives

The primary objective of this trial is to investigate the efficacy of an individually tailored, theory-based motivational counseling intervention on reducing daily sitting time in sedentary patients with RA. The secondary objectives are to explore whether a reduction in daily sitting time is related to reduction in pain and fatigue, improved health-related quality of life (HR-QoL), self-reported physical function, self-efficacy and improved cardiovascular biomarker levels, and to assess the cost-effectiveness of the intervention.

## Methods

### Trial design

The current study is part of the ‘Joint Resources’ research program devoted to health promotion studies in patients with RA [30,31] and initiated in 2009 at Glostrup Hospital. The study is designed as a randomized, controlled, observer-blinded trial with two parallel groups and a primary endpoint of changes in objectively measured daily sitting time. Patient-reported outcomes and other measured variables will be collected before randomization, soon after the intervention (after 16 weeks), and 6 and 18 months post-intervention. Randomization will be performed with a 1:1 allocation (blocks of 10 patients) [32].

### Study setting

Patients will be recruited from lists of RA patients from the rheumatology outpatient clinic at Copenhagen University Hospital, Glostrup, the Capital Region of Denmark (approximately 2,000 patients per year).

### Eligibility criteria

#### Inclusion criteria

Patients can be included in the study if they have been diagnosed with RA (defined by the American College of Rheumatology (ACR) 1987 criteria [33]), are over the age of 18, have self-reported sitting time of 5 hours or more per day (measured by Physical Activity Scale), (PAS version 2.1) [34] have a physical function score < 2.5 (measured by Health Assessment Questionnaire, HAQ [35]), are able to give informed consent, understand and speak Danish, and have access to a mobile phone.

#### Exclusion criteria

Patients will be excluded from the study if they have severe physical disability (HAQ score > 2.5) that would prevent them from reducing daily sitting time (for example, use of wheelchair); are pregnant; participate in vigorous physical activity in their leisure time for more than 8 hours a week (measured by PAS 2.1). Excluded patients or eligible ones who do not want to participate will be registered in one of the following three categories: 1) not meeting the inclusion criteria, 2) refused to participate, or 3) other reasons [36].

### Recruitment, screening and enrollment

**Figure 1:**
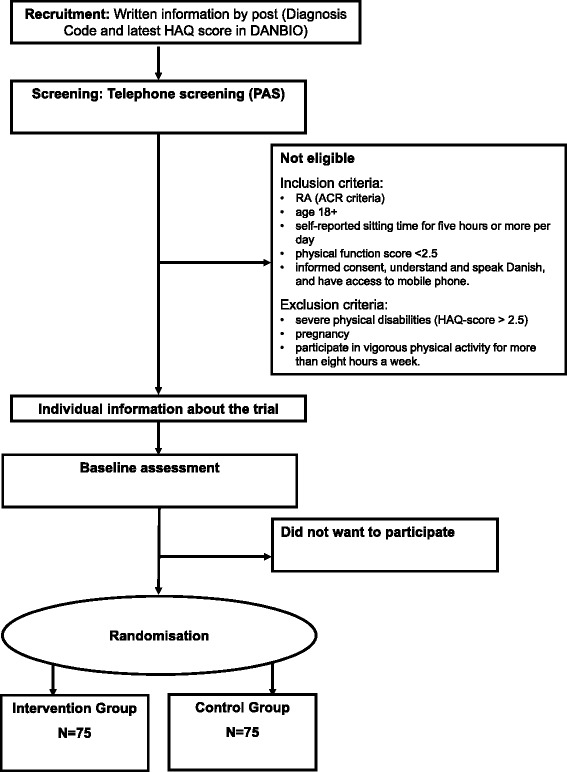
**Recruitment, screening, enrollment, and randomization.** Figure 1 illustrates the procedure of recruitment, screening, enrollment and randomization of patients in the trial.

The patients will be recruited from the Danish National Board of Health biological therapies (DANBIO) database, a nationwide registry that has included > 30,000 patients with inflammatory arthritis in a longitudinal observational cohort [37]. The patients will be screened for diagnosis code and the latest HAQ score in DANBIO (Figure 1). In accordance with the guidelines of the Danish Research Ethics Committee, potentially eligible patients will receive a letter with an invitation to participate, an information leaflet and a folder with basic information about trials and personal rights. A few days later the Project Leader (TT) will contact them by telephone and inquire whether they are interested in participating in the study. If interested, patients answer two ‘screening questions’ on 1) self-reported daily sitting time during leisure time and work, and 2) self-reported participation in vigorous physical activity (both questions are from the Physical Activity Scale (PAS 2.1)) [34] to assess eligibility. If eligible, the patients will be invited to an information session with the Project Leader. It will be possible for the patients to sign the consent form immediately following the session or during the following 2 days. Informed consent will be obtained from each patient prior to the trial.

### Feasibility study and power calculation

A feasibility study has been conducted with the aim of testing and evaluating selected methods and instruments for this trial, as well as testing all elements of the planned study (November 2012 to May 2013). The feasibility study included 19 patients with RA with an average daily sitting time at baseline of 10.13 ± 1.7 hours (mean ± SD). The SD for calculation of sample size in the current trial was based on ActivPAL® data from the feasibility study measured as total daily sitting time. We expect a reduction in average daily sitting time of 50 minutes in the intervention group. This estimated reduction in sitting time is based on a US pilot study from 2012 on obese office workers who reduced their daily sitting time by 48 minutes as a result of a behavioral intervention [28]. For a 2-sample pooled *t*-test of a normal mean difference with a 2-sided significance level of 0.05, assuming a common standard deviation of 102 minutes, a sample size of 67 RA patients per group is required to obtain a power of at least 80% to detect a group mean difference of 50 minutes. Enrolling a sample size of 75 in the intention-to-treat (ITT) population per group has a reasonable power (84.7%) to detect a mean difference of 50 minutes (SAS Power and Sample Size, v. 3.1; SAS Institute Inc., Cary, NC, USA).

### Randomization and blinding

Immediately following the baseline measurements, randomization to either (A) the intervention group (N = 75) or (B) the control group (N = 75) will be performed via computer-generated ‘random numbers’ for each of the 2 groups (blocks of 10 patients). Patients will be informed by TT about their group allocation 1 week after baseline measurements. Patients will not be blinded to the intervention; however, all measurements will be conducted by two occupational therapists who will be blinded to group allocation.

### Intervention and control group

#### Intervention

The intervention includes: 1) Individual motivational counseling including hand-outs of 4 key messages regarding reduction of daily sitting time [38] in combination with 2) Individual Short Text Message Service (SMS) reminders.

1. Individual motivational counseling

The intervention consists of 3 individual motivational counseling sessions (60 to 90 minutes), conducted by one of the project staff (4 nurses or occupational therapists), who have been trained and calibrated in motivational interviewing (MI) techniques [38] for this specific intervention.

Counselors will simultaneously be trained and supervised by a psychologist to ensure that the intervention will be delivered in a similar way based on the theoretical understanding of MI. The first counseling will take place immediately after randomization to the intervention group, the second will be 2 weeks after, and the third 10 weeks after the first counseling (Figure 1). The motivational counseling sessions will take place in an undisturbed room at the hospital.

#### Contents of motivational counseling

The intervention will focus on individual goal setting and self-efficacy, where participants describe their everyday life in terms of sitting time and decide how to reduce it, how to overcome barriers and what behavioral goal, in terms of reducing sitting time, they aim for [39]. Motivational counseling techniques will be used [38].

First, the project staff will introduce the patients to the possible benefits of reducing their daily sitting time. Secondly, they will ask the patients to describe their typical day in order to identify resources and barriers for reduction of daily sitting time and to discuss possible solutions to overcome those barriers and to use the available resources. Thirdly, the patients will set their own behavioral goals for reduction of daily sitting time using a ‘catalogue of ideas’, which contains proposals to reduce and create breaks in sitting time. Specific proposals to reduce daily sitting time are, for example, to stand up during phone conversations or TV-watching, to get up frequently to go to the printer, to get up and walk around at least once every half hour, to get up to change the TV channel, to make only one cup of coffee at a time. During the second and third motivational counseling sessions patients’ own behavioral goals will be evaluated, new goals will be set and possible solution strategies will be discussed. The patients’ motivation and confidence in their own ability to change will be in focus during the last two sessions.

In addition to the motivational counseling, four key messages or themes including ideas and suggestions for reduction of sitting time (that are written in booklets) will be handed to the patients at each session. The 4 key messages are: 1) reduce daily TV-viewing, 2) substitute sitting with standing when possible - at work and/or at home, 3) break up prolonged sitting - by standing up frequently and 4) maximum 30 minutes of sitting per episode [40]. All intervention group participants will fill out a questionnaire on process, side-effects, quality and impact of the intervention when the intervention program is completed.

2) Individual SMS reminders

Based on the patients’ own individual behavioral goals the patients will decide how many weekly SMS reminders they want to receive during the 16-week intervention period and at what time of the day. Examples of SMSs are shown below:

- Hello X. Stand up and allow gravity to assist you to digest your lunch. Bonus: you burn more energy when you stand
- Hi X. Regard vacuum cleaning as a free fitness hour. Make a playlist, put music in your ears and do not stop until the list and the cleaning are done
- Hi X. You have some truly privileged colleagues who will be able to see you stand up by your table this afternoon. Show them how to do it, and they might follow your good example
- Hey X. Put the remote control next to the TV if you turn on the television today. Every time you change the channel your entire body gets some exercise

#### Control group

The control group will be encouraged to maintain their usual lifestyle during the 16-week intervention period. When the last follow-up examination is completed they will be offered the opportunity to join an information session (in groups) about the principles of the intervention.

### Primary outcome measure

Change in total daily sitting time in minutes [41,42] from baseline to 16 weeks.

#### Changes in objectively measured sitting time

Measurements will be obtained using an ActivPAL® 3 TM Activity Monitor (PAL Technologies, Glasgow, UK). This is a small (2.0 × 1.4 × 0.3 inches) and light (20.1 grams) uniaxial accelerometer-based device [43] that is worn anteriorly on the upper right thigh and kept in place by waterproof dressing and adhesive tape. The monitor registers total physical activity level over a 7-day period. The monitor uses accelerometer-derived information about thigh position to estimate time spent in different body positions (that are sitting/lying, standing and walking; sleeping time will be deducted from data). Data will be processed as 0.1 second events data using the ActivPAL® software (version 6.4.1; PAL Technologies, Glasgow, UK). The ActivPAL® monitor has previously been validated against direct observation and compared with the Actigraph® GT3X Activity Monitor accelerometer (Pensacola, FL, USA) [42,44]. It is currently considered the best choice for objective measurement of sitting/lying. The ActivPAL® has also been found sensitive to change in sitting time [44].

### Secondary outcome measures

#### Changes in self-reported sitting time

Changes in self-reported daily sitting time at work and during leisure time will be measured by the Physical Activity Scale 2.1 (PAS 2.1) [34], a modified version of the original PAS questionnaire, which has previously been validated against accelerometer, physical activity logs and maximum oxygen uptake [45,46]. Respondents will be asked to specify number of hours and minutes in an average 24-hour day spent sitting at work and during leisure time.

##### Fatigue

The 20-item Multi-dimensional Fatigue Inventory (MFI 20) [47] will be included to measure fatigue. MFI 20 consists of 20 statements such as ‘I feel enthusiastic’ and classifies fatigue in five dimensions: 1) general fatigue 2) physical fatigue 3) mental fatigue 4) reduced activity 5) reduced motivation.

##### Pain

The Visual Analogue Scale (VAS) [48] transforms the subjective experience of pain to a measurable quantity. The participant indicates his or her pain by putting a mark on the line where the ends are marked with ‘no pain’ to ‘worst imaginable pain’. VAS is included in the HAQ (see below).

##### Physical function

Health Assessment Questionnaire (HAQ) [35] is an instrument that contains twenty items with four possible answers in eight categories of functions within the ADL (regular daily activities): dressing, rising from a seat, eating, walking, personal hygiene, stretching for an object, grabbing objects and everyday activities. In addition, the HAQ includes VAS scales pain, fatigue and general health.

##### Health-related quality of life (HR-QoL)

HR-QoL is assessed with: 1) SF-36 (The Short Form [36] Health Survey) [49] which will be included to measure QoL, which is a generic instrument consisting of 36 items divided into 8 scales: physical function, physical activity limitations, pain, general health, vitality, social function, emotional activity limitations and mental health [49]; 2) EuroQol (EQ-5D-5 L) is a standardized 5-item instrument developed to provide a simple, generic measure of HR-QoL for clinical and economic appraisal [50]. The EQ-5D-5 L descriptive system comprises the following 5 dimensions: mobility, self-care, usual activities, pain/discomfort and anxiety/depression. Each dimension has five levels: no problems, slight problems, moderate problems, severe problems and extreme problems. The responses can be scored into a single utility index based on Danish preference data [51].

##### Self-efficacy

The General Self-Efficacy Scale (GSES) [52] assesses a general sense of perceived self-efficacy with the aim of predicting coping with daily hassles and adaptation after experiencing stressful life events. The instrument is a 10-item scale with statements such as ‘I can usually handle whatever comes my way’ and ‘I am confident that I could deal efficiently with unexpected events’. The response format is ’not at all true’, ‘hardly true’, ‘moderately true’, ‘exactly true’. The instrument is translated and validated in a Danish context.

##### Additional questions on sedentary behavior

Additional questions on duration of sitting time and interruptions in sitting time on weekdays and in weekends are included. The questions have been validated against Actigraph (Pensacola, FL, USA) accelerometers in a recent Danish Study [53].

### Self-administered retrospective questionnaire

At termination of the objective diurnal measurements, a self-administered questionnaire is applied regarding specific recall of time sitting during the measurement period. Time spent sitting during working hours is obtained by the question: ‘during working hours in the measuring period, in average per day, how long time did you spent sitting?’ [5-7,11] reported in hours and minutes. The longest continuous time with uninterrupted sitting is obtained from the question: ‘during working hours in the measuring period, how long is the longest uninterrupted time you have spent sitting?’ reported in hours and minutes. Both questions are also asked for leisure time before/after a workday and leisure time on a complete leisure day [7,11]. The questions used in this study are inspired both by International Physical Activity Questionnaire (IPAQ) [11] and Medical Outcomes Study Patient Assessment Questionnaire MOSPAQ [5,24] (modified Occupational Physical Activity Questionnaire (OPAQ) to specially measure sitting time) together with the new possibilities of the improved objective measurements, that allow specific measurement of sitting during work and leisure time.

#### Anthropometric measures

Height is measured without shoes to the nearest centimeter; weight is measured in light clothing without shoes to the nearest 0.1 kg (OBH Nordica, Slim Light, 150 kg; Taastrup, Denmark); waist circumference is measured midway between the lower rib margin and the iliac crest to the nearest 0.5 centimeter, without any pressure to the skin and with an unstretched tape measure; hip circumference is measured at the point yielding the maximum circumference over the buttocks to the nearest 0.5 centimeter. The tape should be held in a horizontal plane touching the skin but not indenting the soft tissue. Body mass index (BMI, kg/m^2^) and waist-hip ratio are calculated.

#### Serum lipids

A venous blood sample will be drawn (not fasting). Total cholesterol, high-density lipoprotein cholesterol (HDL), and triglycerides will be measured by an enzymatic method on the Vitros 5.1 FS from Ortho Clinical Diagnostics (Birkerød, Denmark). Low-density lipoprotein cholesterol (LDL) and very low-density lipoprotein cholesterol (VLDL) will be calculated by the formulae:

$$ VLDL = Triglyceride \times 0.45 $$

$$ LDL = Total\  cholesterol - HDL + VLDL $$

In addition, C-reactive protein (CRP) and glycosylated hemoglobin, type A1c (HbA1c) will be measured on Vitros 5.1 FS (Birkerød, Denmark) and G8 HPLC Analyzer from TOSOH (Aarhus, Denmark).

#### Blood pressure

Blood pressure will be measured, after 5 to 10 minutes of rest, 3 times at the right upper arm (average of the 3 measurements) with the participant in a sitting/lying position.

#### Additional information

In addition, information regarding demographics, lifestyle, time of diagnosis, consumption of pain killers and comorbidity (which are diabetes, hypertension, heart attack, stroke, chronic obstructive pulmonary disease (COPD), cancer, osteoarthritis, osteoporosis, asthma and depression) will be obtained; all self-reported. Information about DAS 28 (Disease Activity Score Based on 28 Joints and 4 variables including CRP [54], actual medical treatment and status of RA (for example, IgM-RF (rheumatoid factor of the immunoglobulin M class and/or ACPA (anti-citrullinated protein antibody)) will be obtained from the DANBIO registry [37] and the medical record.

#### Cost data

For the analysis of intervention costs, activities related to the intervention will be registered for each individual patient by the health care providers. In addition, data will be collected on time use related to each activity and relevant unit cost of resources. Other resource use and costs related to health care provisions will be obtained for each patient from the national health registries for hospitals, primary care, and purchase of prescription medication will be obtained from primary care pharmacies. These data will be obtained from the National Board of Health. Due to the confidentiality of these data (prescription data) they will be stored and analyzed through Statistics Denmark’s Research computer.

### Participant timeline

The trial will last 22 months including a 16-week intervention, and 6 and 18 months follow-up (Figure 1).

### Data collection

Measurements will be repeated 4 times during the 22 months: 1) at baseline; 2) at the end of the intervention (16 weeks after intervention start); 3) 6 months post intervention and finally (4) 18 months post intervention (Table 1). At each time point patients will complete self-reported questionnaires regarding demographics, lifestyle, sitting time at work and in leisure time, physical activity, physical function, pain, fatigue, health-related quality of life (HR-QoL) and general self-efficacy. Blood pressure, height, weight, waist circumference will be measured subsequently. Blood samples will be drawn and stored at the Department of Diagnostics, Division of Clinical Biochemistry, Glostrup Hospital, University of Copenhagen.

**Table 1:** **Collection of patient characteristics and outcome measures**

	**Screening**	**Baseline**	**4 months by end of intervention**	**6 months post intervention**	**18 months post intervention**
Background					
Marital status		X			
Age		X			
Country of birth		X			
Cohabiting		X			
Level of education		X			
Employment status		X			
Annual household income		X			
Health-related issues					
Smoking		X	X	X	X
Alcohol consumption		X	X	X	X
Medical history					
Diagnosed with RA	X				
Actual treatment for RA		X	X	X	X
DAS 28		X	X	X	X
IgM-Rheumatoid Factor		X			
ACPA		X			
Co-morbidities					
Diabetes		X	X	X	X
Hypertension		X	X	X	X
Heart attack		X	X	X	X
Stroke		X	X	X	X
COPD		X	X	X	X
Cancer		X	X	X	X
Osteoarthritis		X	X	X	X
Osteoporosis		X	X	X	X
Asthma		X	X	X	X
Depression		X	X	X	X
Medicine					
Consumption of painkillers		X	X	X	X
Serum lipids					
Total cholesterol		X	X	X	X
High-density lipoprotein cholesterol (HDL)		X	X	X	X
Triglycerides		X	X	X	X
Low-density lipoprotein cholesterol (LDL)		X	X	X	X
Very low-density lipoprotein cholesterol (VLDL)		X	X	X	X
C-reactive protein (CRP)		X	X	X	X
HbA1c		X	X	X	X
Anthropometric measures					
Weight		X	X	X	X
Height		X			
Blood pressure		X	X	X	X
Waist circumference		X	X	X	X
Body mass index; BMI		X	X	X	X
Daily sitting time					
Measured total sitting time in hours and minutes (measured by ActivePAL®)		X	X	X	X
Self-reported daily sitting time (measured by physical activity - PAS, item 4)	X	X	X	X	X
Questionnaires (self-reported)					
Physical Activity Scale - PAS	X	X	X	X	X
Fatigue - MFI		X	X	X	X
Pain - VAS		X	X	X	X
Physical function - HAQ	X	X	X	X	X
HR-QoL - SF-36		X	X	X	X
HR-QoL - EuroQol/EQ-5D		X	X	X	X
General Self-Efficacy Scale - GSES		X	X	X	X
Specific questions on sedentary behavior at work and leisure		X	X	X	X

Table 1 shows the procedure of collecting data (objectively measured and self-reported) about the trial patients from baseline to 18 months follow-up.

Anti-CCP, anti-citrullinated peptide antibody; DAS 28: Disease Activity Score Based on 28 Joints; GSES: The General Self-Efficacy Scale; HAQ: Health Assessment Questionnaire; HR-QoL, health-related quality of life; MFI: Multi-dimensional Fatigue Inventory; RA: rheumatoid arthritis; SF-36: The Short Form [36] Health Survey.

Baseline demographic characteristics of patients are collected (electronically) via a self-report questionnaire (using tablets). Two occupational therapists will attach the ActivPAL® monitor [41] and instruct patients in how to wear the monitor 24 hours per day for 7 days. All ActivPAL® will be coded to start from midnight following the first day of application. One week (7 days) after baseline measurements the patient will return to the hospital for removal of the ActivPAL®. At the same time the patient will be informed by the Project Leader (TT) about the group allocation. One week after each of the follow-up measurements the patients will return the ActivPAL® in a closed envelope. During each 7 day-period with ActivPal® the patients fill a diary about their resting time and sleeping time in order to isolate their sitting time from sleeping time. At each medical visit to the outpatient clinic, the treating rheumatologist registers information in DANBIO including data regarding disease characteristics (for example, patient-reported outcomes, disease activity, functional status, disease course) and medical treatment [37]. Patients will not be blinded to the intervention; however, all assessors will be blinded for the two participating groups.

### Data management

Since all data will be supplied directly by the patients and the assessors through an online interface via a tablet there is no risk of data loss or distortion along the way. Data will be stored encrypted and in unidentifiable form (using participant-numbers) in the server at Glostrup Hospital, University of Copenhagen. Standard missing data analysis will determine whether or not unexpected but missing data due to participant drop-out are random.

### Statistical analysis

The scoring of the standardized questionnaires will be carried out according to the guidelines from instrument developers. All data analyses will be carried out according to a pre-established analysis plan; all analyses will be done applying SAS software (v. 9.3; SAS Institute Inc., Cary, NC, USA). All descriptive statistics and tests will be reported in accordance with the recommendations of the ‘Enhancing the QUAlity and Transparency Of health Research’ (EQUATOR) network: the CONSORT statement [55]. In order to evaluate the empirical distributions of the continuous outcomes, visual inspection will be used to suggest whether the assumption of normality is reasonable. The univariate procedure (PROC UNIVARIATE) statement will be used for summarizing the data. All analyses will be conducted according to the ITT principle; that is analyzing participant outcomes according to the group to which they are randomized, even if some participants do not receive the intervention according to the randomization. We will run the analyses where missing data are imputed by multiple imputation. For sensitivity we will also perform analyses ‘as observed’ analyses where we will not use any missing data imputation. All reported *P*-values and 95% confidence intervals (95% CI) will be 2-sided and will not be adjusted for multiple comparisons. Unless stated otherwise, results will be expressed as the difference between the group means and 95% CI with the associated *P*-values, based on a general linear model (GLM). Data will be analyzed using a one-factor ‘Analysis of Covariance’ (ANCOVA), with a factor for Group, using the baseline value as covariate to reduce the random variation and increase the statistical power.

Proportions will be compared by estimating the risk difference with 95% CIs for each dichotomous outcome; including a Wald-Z-test for the null hypothesis that there is no difference between the proportions.

Secondly, to relate the results to compliance, a ‘per protocol’ analysis will also be used. The ‘per protocol’ population is defined as the patients who have ‘completed’ the intervention to which they were allocated according to the principles described in the intervention section above.

### Cost analysis

The cost analysis will report the resource use and cost of the intervention. For the intervention and control group, survey and registry-based data on resource use, including services provided in the health care sector (primary and secondary care) and pharmaceuticals, will be aggregated for each individual in intervals determined by the time of data collection. Cost data will be interpolated to total cost for the whole observation period (18 months). The aggregated cost data will be analyzed as costs attributable to the intervention (that is difference in difference) and reported as mean values with 95% (bootstrap) CIs. Due to the non-normal distribution of cost data generalized linear models will be employed with appropriate choice of link function.

The analysis of cost-effectiveness will compare the observed gain in Quality-Adjusted Life Years (QALYs) (HR-QoL over 18 months) with the observed incremental cost (over 18 months) by estimating incremental cost-effectiveness ratios (ICER). To express statistical uncertainty in the ICER estimates acceptability curves will be developed to express the likelihood of the intervention being cost-effective at various QALY threshold values.

## Monitoring

### Ethics, confidentiality and dissemination

The trial will be performed in accordance with the Helsinki Declaration. The project has been approved by the Regional Committee on Biomedical Research Ethics September 2012 as registry-based research (REC; reference number, H-2-2012-112, document number, 37276). The study was approved by the Danish Data Protection Agency (ref number 711-1-08). All formal and safety rules in the process from data collected for publication are observed (Danish Data Protection Agency, Research Ethics Committee, Clinicaltrial.gov, information technology (IT) issues, and so on). Anyone who has access to the full relevant data sets (chief executive, all supervisors, and project staff) is familiar with the formal and safety rules. All information collected during the course of the study will be kept strictly confidential in accordance with Danish Data Protection Agency rules; operationally, this will include consent from the patients.

It is planned to publish at least four scientific papers based on the trial in peer-reviewed journals.

In addition, the following sources are included in the communication plan:

- Papers (peer-reviewed and popular science)
- Presentations at conferences (national and international) or similar
- Collaboration with the Danish Rheumatism Association - both members and groups of employees
- Other relevant patient organizations covering patients with musculoskeletal diseases, and press

### Access to data

RC will analyze data in collaboration with TT, BAE, MA. JS will analyze the cost-effectiveness data. The project group will have access to data as well as to the DANBIO registry [37,56]. The patient data in DANBIO are protected by a unique log-in for each user. Data are encrypted and logged. Data generated in the trial belong to Glostrup Hospital, University of Copenhagen. The steering group ‘Joint Resources’ will be involved in the case of query about access to data.

## Discussion

This trial protocol describes the design of an innovative randomized controlled trial, which aims to test whether sedentary behavior can be reduced in patients with RA through behavioral lifestyle change. To the best of our knowledge, this is the first randomized trial to investigate the effect of an individually tailored motivational counseling intervention on daily sitting time in patients with RA. The intervention is developed and based upon results from studies in population-based samples of sedentary adults and in elderly and obese sedentary populations. The population under study, men and women with RA, represents a group of patients with chronic disease and increased risk of developing diabetes and cardiovascular diseases. In addition, a large proportion of patients with RA struggle with strained joints and immobility in daily life. If effective, the intervention may reduce the risk of cardiovascular disease and type 2 diabetes in patients with RA by reducing their daily sitting time. It should be noted that a possible reduction in sitting time, the primary outcome, does not necessarily imply a reduction in cardiovascular disease (CVD) risk. However, a possible effect of the intervention on secondary outcomes, CVD biomarkers (cholesterol and so on) will be simultaneously registered and studied.

We cannot rule out that wearing an ActivPAL® may cause reactivity and as such in itself act as a ‘behavioral intervention’. However, participants in both groups are measured at baseline and follow-up, and the size of the between arm difference is, therefore, not likely to be affected or considered in the sample size calculation. Also ActivPAL® monitors start measuring from midnight following the first day of application in order to minimize reactivity by allowing participants to get used to wearing the device. Finally, the ActivPAL® measurements are not shown to participants until after the study is completed.

It should be noted that in behavioral intervention RCTs patients are difficult to blind. We could have offered the control group participants a ‘sham’ counseling intervention with counseling on something else than reduction of sedentary behavior. However, this could potentially induce an effect in the control group which we wanted to avoid.

We expect the intervention to result in reduced pain (less use of painkillers), reduced fatigue, increased daily physical functioning, improved self-efficacy and better general health status. The intervention is simple and could relatively easily be implemented in clinical practice along with lifestyle advice and recommendations on physical activity, sports and exercise, diet and smoking habits. In addition, we expect the intervention method to be transferable to other groups of chronically ill individuals with prolonged sitting time and mobility limitations.

## Trial status

Recruitment for the trial is planned to start in April 2013 and will end in December 2014.

## Abbreviations

ACPA: anti-citrullinated protein antibody
ACR: American College of Rheumatology
ANCOVA: Analysis of Covariance
BMI: body mass index
COPD: chronic obstructive pulmonary disease
CRP: C-reactive protein
CVD: cardiovascular disease
DANBIO: Danish National Board of Health biological therapies database
DAS 28: Disease Activity Score Based on 28 Joints
EQUATOR: Enhancing the QUAlity and Transparency Of health Research
EQ-5D-5 L: EuroQol
GLM: general linear model
GSES: The General Self-Efficacy Scale
HAQ: Health assessment questionnaire
HbA1c: glycosylated hemoglobin, type A1c
HR-QoL: health-related quality of life
ICER: incremental cost-effectiveness ratios
IgM-RF: rheumatoid factor of the immunoglobulin M class
IPAQ: International Physical Activity Questionnaire
IT: information technology
ITT: intention-to-treat
LDL: low-density lipoprotein cholesterol
METs: Metabolic Equivalent of Task
MFI: Multi-dimensional Fatigue Inventory
MI: motivational interviewing
MOSPAQ: Medical Outcomes Study Patient Assessment Questionnaire
MVPA: moderate and vigorous physical activity
OPAQ: Occupational Physical Activity Questionnaire
PAS: Physical Activity Scale
PROC UNIVARIATE: univariate procedure
QALYs: Quality-Adjusted Life Years
RA: rheumatoid arthritis
SF-36: The Short Form Health Survey
SMS: Short Text Message Service
VAS: The Visual Analogue Scale
VLDL: very low-density lipoprotein cholesterol

## References

[1] Kohl HW, Craig CL, Lambert EV, Inoue S, Alkandari JR, Leetongin G (2012). The pandemic of physical inactivity: global action for public health. Lancet.

[2] WHO. Global health risks. Mortality and burden of disease attributable to selected major risks. World Health Organisation;2009.

[3] Ford ES, Caspersen CJ (2012). Sedentary behaviour and cardiovascular disease: a review of prospective studies. Int J Epidemiol.

[4] Grontved A, Hu FB (2011). Television viewing and risk of type 2 diabetes, cardiovascular disease, and all-cause mortality: a meta-analysis. JAMA.

[5] Wilmot EG, Edwardson CL, Achana FA, Davies MJ, Gorely T, Gray LJ (2012). Sedentary time in adults and the association with diabetes, cardiovascular disease and death: systematic review and meta-analysis. Diabetologia.

[6] John H, Toms TE, Kitas GD (2011). Rheumatoid arthritis: is it a coronary heart disease equivalent?. Curr Opin Cardiol.

[7] Kerola AM, Kerola T, Kauppi MJ, Kautiainen H, Virta LJ, Puolakka K (2013). Cardiovascular comorbidities antedating the diagnosis of rheumatoid arthritis. Ann Rheum Dis.

[8] Metsios GS, Stavropoulos-Kalinoglou A, van Zanten JJV, Treharne GJ, Panoulas VF, Douglas KM (2008). Rheumatoid arthritis, cardiovascular disease and physical exercise: a systematic review. Rheumatology (Oxford).

[9] Sokka T, Hakkinen A, Kautiainen H, Maillefert JF, Toloza S, Mork Hansen T (2008). Physical inactivity in patients with rheumatoid arthritis: data from twenty-one countries in a cross-sectional, international study. Arthritis Rheum.

[10] Hewlett S, Sanderson T, May J, Alten R, Bingham CO, Cross M (2012). ‘I’m hurting, I want to kill myself’: rheumatoid arthritis flare is more than a high joint count - an international patient perspective on flare where medical help is sought. Rheumatology (Oxford).

[11] Kett C, Flint J, Openshaw M, Raza K, Kumar K (2010). Self-management strategies used during flares of rheumatoid arthritis in an ethnically diverse population. Musculoskeletal Care.

[12] Hurkmans E, van der Giesen FJ, Vliet Vlieland TP, Schoones J, Van den Ende EC (2009). Dynamic exercise programs (aerobic capacity and/or muscle strength training) in patients with rheumatoid arthritis. Cochrane Database Syst Rev.

[13] Cairns AP, McVeigh JG (2009). A systematic review of the effects of dynamic exercise in rheumatoid arthritis. Rheumatol Int.

[14] Wilcox S, Der Ananian C, Abbott J, Vrazel J, Ramsey C, Sharpe PA (2006). Perceived exercise barriers, enablers, and benefits among exercising and nonexercising adults with arthritis: results from a qualitative study. Arthritis Rheum.

[15] Sedentary Behaviour Research N (2012). Letter to the editor: standardized use of the terms ‘sedentary’ and ‘sedentary behaviours’. Appl Physiol Nutr Metab.

[16] Bauman A, Ainsworth BE, Sallis JF, Hagstromer M, Craig CL, Bull FC (2011). The descriptive epidemiology of sitting. A 20-country comparison using the International Physical Activity Questionnaire (IPAQ). Am J Prev Med.

[17] Matthews CE, Chen KY, Freedson PS, Buchowski MS, Beech BM, Pate RR (2008). Amount of time spent in sedentary behaviors in the United States, 2003–2004. Am J Epidemiol.

[18] Healy GN, Matthews CE, Dunstan DW, Winkler EA, Owen N (2011). Sedentary time and cardio-metabolic biomarkers in US adults: NHANES 2003–06. Eur Heart J.

[19] Rand D, Eng JJ, Tang PF, Jeng JS, Hung C (2009). How active are people with stroke?: use of accelerometers to assess physical activity. Stroke.

[20] Cavanaugh JT, Coleman KL, Gaines JM, Laing L, Morey MC (2007). Using step activity monitoring to characterize ambulatory activity in community-dwelling older adults. J Am Geriatr Soc.

[21] Chastin SF, Baker K, Jones D, Burn D, Granat MH, Rochester L (2010). The pattern of habitual sedentary behavior is different in advanced Parkinson’s disease. Mov Disord.

[22] Prioreschi A, Hodkinson B, Avidon I, Tikly M, McVeigh JA (2013). The clinical utility of accelerometry in patients with rheumatoid arthritis. Rheumatology (Oxford).

[23] Almeida GJ, Wasko MC, Jeong K, Moore CG, Piva SR (2011). Physical activity measured by the SenseWear Armband in women with rheumatoid arthritis. Phys Ther.

[24] Paul L, Rafferty D, Marshall-McKenna R, Gill JM, McInnes I, Porter D (2014). Oxygen cost of walking, physical activity, and sedentary behaviours in rheumatoid arthritis. Scand J Rheumatol.

[25] Prioreschi A, Hodkinson B, Tikly M, McVeigh JA (2014). Changes in physical activity measured by accelerometry following initiation of DMARD therapy in rheumatoid arthritis. Rheumatology (Oxford).

[26] Manns PJ, Dunstan DW, Owen N, Healy GN (2012). Addressing the nonexercise part of the activity continuum: a more realistic and achievable approach to activity programming for adults with mobility disability?. Phys Ther.

[27] Gardiner PA, Eakin EG, Healy GN, Owen N (2011). Feasibility of reducing older adults’ sedentary time. Am J Prev Med.

[28] Kozey-Keadle S, Libertine A, Staudenmayer J, Freedson P (2012). The feasibility of reducing and measuring sedentary time among overweight, non-exercising office workers. J Obes.

[29] Otten JJ, Jones KE, Littenberg B, Harvey-Berino J (2009). Effects of television viewing reduction on energy intake and expenditure in overweight and obese adults: a randomized controlled trial. Arch Intern Med.

[30] Loeppenthin K, Esbensen B, Ostergaard M, Jennum P, Thomsen T, Midtgaard J (2014). Physical activity maintenance in patients with rheumatoid arthritis: a qualitative study. Clin Rehabil.

[31] Loppenthin K, Esbensen BA, Jennum P, Ostergaard M, Christensen JF, Thomsen T (2014). Effect of intermittent aerobic exercise on sleep quality and sleep disturbances in patients with rheumatoid arthritis - design of a randomized controlled trial. BMC Musculoskelet Disord.

[32] Chan AW, Tetzlaff JM, Gotzsche PC, Altman DG, Mann H, Berlin JA (2013). SPIRIT 2013 explanation and elaboration: guidance for protocols of clinical trials. BMJ.

[33] Arnett FC, Edworthy SM, Bloch DA, McShane DJ, Fries JF, Cooper NS (1988). The American rheumatism association 1987 revised criteria for the classification of rheumatoid arthritis. Arthritis Rheum.

[34] Andersen LG, Groenvold M, Jorgensen T, Aadahl M (2010). Construct validity of a revised physical activity scale and testing by cognitive interviewing. Scand J Public Health.

[35] Bruce B, Fries JF (2003). The Stanford health assessment questionnaire: a review of its history, issues, progress, and documentation. J Rheumatol.

[36] Schulz KF, Altman DG, Moher D (2011). CONSORT 2010 statement: updated guidelines for reporting parallel group randomised trials. Int J Surg.

[37] Hetland ML (2011). DANBIO - powerful research database and electronic patient record. Rheumatology (Oxford).

[38] Rollnick S, Miller WR, CC. B. Motivational interviewing in health care - helping patients change behaviour. The Guildford Press;2008.

[39] Bandura A (1986). Social foundation of thoughts and action: a social cognitive theory.

[40] Epstein LH, Roemmich JN (2001). Reducing sedentary behavior: role in modifying physical activity. Exerc Sport Sci Rev.

[41] Godfrey A, Culhane KM, Lyons GM (2007). Comparison of the performance of the ActivPAL professional physical activity logger to a discrete accelerometer-based activity monitor. Med Eng Phys.

[42] Grant PM, Ryan CG, Tigbe WW, Granat MH (2006). The validation of a novel activity monitor in the measurement of posture and motion during everyday activities. Br J Sports Med.

[43] PAL Technologies. Providing the evidence. Glasgow 2014. http://www.paltechnologies.com. Cited 23 May 2014.

[44] Kozey-Keadle S, Libertine A, Lyden K, Staudenmayer J, Freedson PS (2011). Validation of wearable monitors for assessing sedentary behavior. Med Sci Sports Exerc.

[45] Aadahl M, Jorgensen T (2003). Validation of a new self-report instrument for measuring physical activity. Med Sci Sports Exerc.

[46] Aadahl M, Kjaer M, Kristensen JH, Mollerup B, Jorgensen T (2007). Self-reported physical activity compared with maximal oxygen uptake in adults. Eur J Cardiovasc Prev Rehabil.

[47] Smets EM, Garssen B, Bonke B, De Haes JC (1995). The Multidimensional Fatigue Inventory (MFI) psychometric qualities of an instrument to assess fatigue. J Psychosom Res.

[48] Price DD, McGrath PA, Rafii A, Buckingham B (1983). The validation of visual analogue scales as ratio scale measures for chronic and experimental pain. Pain.

[49] Alonso J, Ferrer M, Gandek B, Ware JE, Aaronson NK, Mosconi P (2004). Health-related quality of life associated with chronic conditions in eight countries: results from the International Quality of Life Assessment (IQOLA) Project. Qual Life Res.

[50] EuroQol - a new facility for the measurement of health-related quality of life. Health Policy. 1990;16(3):199–208. 10.1016/0168-8510(90)90421-9 10109801

[51] Wittrup-Jensen KU, Lauridsen J, Gudex C, Pedersen KM (2009). Generation of a Danish TTO value set for EQ-5D health states. Scand J Public Health.

[52] Luszczynska A, Scholz U, Schwarzer R (2005). The general self-efficacy scale: multicultural validation studies. J Psychol.

[53] Lagersted-Olsen J, Korshoj M, Skotte J, Carneiro IG, Sogaard K, Holtermann A. Comparison of objectively measured and self-reported time spent sitting. Int J Sports Med. 2013. 10.1055/s-0033-1358467 24258469

[54] The Disease Activity Score (DAS). Nijmegen: Radboud University Nijmegen, Medical Centre. 2014. http://www.das-score.nl. Cited 23 May 2014.

[55] Moher D, Hopewell S, Schulz KF, Montori V, Gotzsche PC, Devereaux PJ (2010). CONSORT 2010 explanation and elaboration: updated guidelines for reporting parallel group randomised trials. BMJ.

[56] DANBIO Database. Glostrup: DANBIO;2014. https://danbio-online.dk. Cited 23 May 2014.

